# Extracorporeal membrane oxygenation for 2009 influenza A(H1N1) severe respiratory failure in Japan

**DOI:** 10.1007/s00540-012-1402-x

**Published:** 2012-05-23

**Authors:** Shinhiro Takeda, Toru Kotani, Satoshi Nakagawa, Shingo Ichiba, Toshiyuki Aokage, Ryoichi Ochiai, Nobuyuki Taenaka, Kaneyuki Kawamae, Masaji Nishimura, Yoshihito Ujike, Kimitaka Tajimi, Committee of Crisis Control, the Japanese Society of Respiratory Care Medicine and Committee of Pandemic H1N1 Surveillance, the Japanese Society of Intensive Care Medicine

**Affiliations:** 1 Department of Anesthesiology and Intensive Care Unit, Nippon Medical School, 1-1-5 Sendagi, Bunkyo-ku, Tokyo, 113-8603 Japan; 2 Department of Anesthesiology and Critical Care, Tokyo Women’s Medical University, 8-1 Kawada-cho, Shinjuku-ku, Tokyo, 162-8666 Japan; 3 Critical Care Medicine, National Center for Child Health and Development, 2-10-1 Okura, Setagaya-ku, Tokyo, 157-8535 Japan; 4 Department of Community and Emergency Medicine, Okayama University Graduate School of Medicine, Dentistry, and Pharmaceutical Sciences, 2-5-1 Shikata-cho, Kita-ku, Okayama, 700-8558 Japan; 5 Intensive Care Unit and Cardiac Care Unit, Nippon Medical School Hospital, 1-1-5 Sendagi, Bunkyo-ku, Tokyo, 113-8603 Japan; 6 The First Department of Anesthesiology, Toho University School of Medicine, 6-11-1 Omori-Nishi, Ota-ku, Tokyo, 143-8541 Japan; 7 Takarazuka City Hospital, 4-5-1 Kohama, Takarazuka, 665-0827 Japan; 8 Department of Anesthesiology, Faculty of Medicine, Yamagata University, 2-2-2 Iida-Nishi, Yamagata, 990-9585 Japan; 9 Emergency and Critical Care Medicine, The University of Tokushima Graduate School, 3-18-15 Kuramoto, Tokushima, 770-8503 Japan; 10 Department of Emergency and Critical Care Medicine, Okayama University School of Medicine and Hospital, 2-5-1 Shikata-cho, Kita-ku, Okayama, 700-8558 Japan; 11 Department of Emergency and Critical Care Medicine, Akita University Graduate School of Medicine, 1-1-1 Hondo, Akita, 010-8543 Japan

**Keywords:** ECMO, Influenza, Respiratory failure, Mortality

## Abstract

**Purpose:**

To evaluate procedures and outcomes of extracorporeal membrane oxygenation (ECMO) therapy applied to 2009 influenza A(H1N1) severe respiratory failure patients in Japan.

**Methods:**

This observational study used database information about adults who received ECMO therapy for H1N1-related severe respiratory failure from April 1, 2010 to March 31, 2011.

**Results:**

Fourteen patients from 12 facilities were enrolled. Anti-influenza drugs were used in all cases. Before the start of ECMO, the lowest PaO_2_/FiO_2_ was median (interquartile) of 50 (40–55) mmHg, the highest peak inspiratory pressure was 30 (29–35) cmH_2_O, and mechanical ventilation had been applied for at least 7 days in 5 patients. None of the facilities had extensive experience with ECMO for respiratory failure (6 facilities, no previous experience; 5 facilities, one or two cases annually). The blood drainage cannula was smaller than 20 Fr. in 10 patients (71.4 %). The duration of ECMO was 8.5 (4.0–10.8) days. The duration of each circuit was only 4.0 (3.2–5.3) days, and the ECMO circuit had to be renewed 19 times (10 cases). Thirteen patients (92.9 %) developed adverse events associated with ECMO, such as oxygenator failure, massive bleeding, and disseminated intravascular coagulation. The survival rate was 35.7 % (5 patients).

**Conclusion:**

ECMO therapy for H1N1-related severe respiratory failure in Japan has very poor outcomes, and most patients developed adverse events. However, this result does not refute the effectiveness of ECMO. One possible cause of these poor outcomes is the lack of satisfactory equipment, therapeutic guidelines, and systems for patient transfer to central facilities.

## Introduction

The World Health Organization reported individuals infected with a novel swine-origin influenza virus 2009 influenza A(H1N1) in Mexico and the United States in April 2009 [1]. This report was quickly followed by a worldwide pandemic. The severity of infection was the same as that of seasonal influenza in many cases, but more than a few patients developed severe respiratory failure of a kind that was unlikely to have resulted from conventional seasonal influenza.

Serious cases in which oxygenation could not be maintained by conventional mechanical ventilation were managed with extracorporeal membrane oxygenation (ECMO), often yielding excellent outcomes. According to reports from Australia and New Zealand, 68 patients received ECMO therapy during the 2-month period at the height of the epidemic, and the survival rate exceeded 70 % [2]. The Extracorporeal Life Support Organization (ELSO) reported a survival rate of more than 60 % for 323 patients [3]. According to a report from the United Kingdom, treatment outcomes in very severe cases were better when ECMO was applied than when only conventional mechanical ventilation was employed [4]. Utilization of the ECMO network system and transfer of patients to the ECMO center were considered to be among the factors that resulted in better treatment outcomes [4–7]. The ECMO Center Karolinska, Sweden, reported a survival rate of more than 90 % [5].

In Japan, however, no network system or center for ECMO therapy is available, and ECMO has been applied only at individual medical facilities in cases where this therapy was indicated. No data are as yet available in Japan regarding the outcomes of ECMO therapy for 2009 influenza A(H1N1). The present study was undertaken to analyze the procedures and outcomes of ECMO therapy applied to adult patients, using information on patients infected with H1N1 and admitted to intensive care units (ICUs). These data were collected by the Committee of Crisis Control, the Japanese Society of Respiratory Care Medicine and the Committee of Pandemic H1N1 Surveillance, the Japanese Society of Intensive Care Medicine.

## Methods

The study involved adults who received ECMO therapy for severe respiratory failure associated with H1N1 influenza from April 1, 2010 to March 31, 2011. A database was created using patient information that had been collected from attending physicians of the facilities participating in this study; the information was provided at the physicians’ own discretion in response to a public notification (data collection on ICU patients infected with H1N1) issued by the Japanese Society of Respiratory Care Medicine and the Japanese Society of Intensive Care Medicine. Informed consent from individual patients was obtained by each reporting physician and facility. Data collection pertaining to the findings before hospitalization and upon admission included age, sex, body weight, body mass index (BMI), body temperature, Acute Physiology and Chronic Health Evaluation (APACHE) II score, underlying disease, and vaccination. During treatment, information was collected about complications, Sequential Organ Failure Assessment (SOFA) score, type of anti-influenza drug, mechanical ventilation, blood gas analysis, and continuous renal replacement therapy. In addition, data were collected about the duration of mechanical ventilation, duration of ICU stay, hospitalization period, and patient outcome.

From this database, adult patients who had received ECMO therapy were extracted for analysis. The physician in charge at each facility that provided the ECMO therapy was requested by e-mail or telephone to supply additional detailed information with regard to the following: the equipment used for ECMO therapy, cannula size, site of approach with the cannula, duration of ECMO therapy, duration of mechanical ventilation before the start of therapy, adverse events, cause of death, and previous ECMO experience.

With respect to the ECMO therapy procedures, detailed information was collected on each survey item. The survival and non-survival groups were compared using the Mann–Whitney test, Fisher’s exact test, or chi-square test. Statistical analyses were performed using SPSS II (Abacus Concepts, Berkeley, CA, USA). All values are reported as median (interquartile), and all *p* values <0.05 are considered statistically significant.

## Results

### Patient background and treatment course (Table 1)

Fourteen patients from 12 facilities were enrolled in this study. The survival rate was as low as 35.7 % (5 patients). Weaning from ECMO was impossible in all the patients who later died.

**Table 1:** Patient background and treatment course

	All cases (14 patients)	Survival group (5 patients)	Non-survival group (9 patients)
Age (years)	54 (43–60)	54 (35–58)	54 (41–62)
Sex (male/female)	12/2	4/1	8/1
Weight (kg)	70 (64–80)	69 (54–86)	70 (64–80)
Obesity			
35 > BMI ≥ 25	7	3	4
BMI ≥ 35	1	0	1
Body temperature (°C)			
At first examination	38.8 (37.1–39.1)	38.8 (36.8–39.0)	38.8 (37.3–39.4)
Maximum	39.4 (38.7–39.8)	39.2 (39.0–39.7)	39.5 (38.1–39.9)
APACHE II score	17 (12–25)	16 (12–24)	17 (12–28)
Predicted death rate (%)	24.9 (14.6–54.1)	23.5 (15.5–49.7)	26.2 (14.6–61.5)
Maximum SOFA score	15.5 (12.0–19.3)	12.0 (10.0–15.0)	19.0 (14.5–20.5)*
Underlying condition			
Drug abuse	1	0	1
Pregnancy	1	1	0
Vaccination (H1N1 + seasonal)	1	0	1
Complications			
Acute renal failure	7	2	5
Acute hepatic failure	3	0	3
Culture-confirmed infection	4	2	2
Shock	4	1	3
Medical treatment			
Peramivir	11	4	7
Oseltamivir	6	2	4
Zanamivir	1	0	1
Antibiotics	14	5	9
Steroid			
High + low dose	6	3	3
High dose	3	0	3
Low dose	2	2	0
Sivelestat	4	1	3
Vasoactive drugs	13	4	9
Rescue therapies and adjunctive therapies			
Prone position	3	1	2
APRV	13	5	8
Nitric oxide	1	0	1
CRRT	7	2	5
Respiratory severity and ventilator parameters			
Lowest PaO_2_/FiO_2_ (mmHg)	50 (40–55)	49 (43–53)	50 (40–60)
Highest PEEP (cmH_2_O)	24 (17–30)	22 (15–28)	28 (18–30)
Highest PIP (cmH_2_O)	30 (29–35)	29 (23–42)	30 (30–35)
Ventilator days (days)	19 (9–25)	24 (16–37)	10 (6–25)
Length of stay in ICU (days)	17 (9–26)	24 (16–31)	10 (6–25)
Hospitalization (days)	25 (12–53)	69 (35–83)	15 (6–25)**

Data expression, median (interquartile)

*BMI* body mass index, *APACHE* Acute Physiology and Chronic Health Evaluation, *SOFA* Sequential Organ Failure Assessment, *DIC* disseminated intravascular coagulation, *APRV* airway pressure release ventilation, *CRRT* continuous renal replacement therapy, *PEEP* positive end-expiratory pressure, *PIP* peak inspiratory pressure, *ICU* intensive care unit

* *p* = 0.004 (survival group vs. non-survival group); ** *p* = 0.036 (survival group vs. non-survival group)

Most patients were male (85.7 %). The mortality rate predicted from the APACHE II score was 24.9 %, but the actual mortality rate (64.3 %) was 2.5 times higher. None of the patients had chronic respiratory failure, chronic heart failure, or immunological diseases as underlying disorders. Anti-influenza drugs were used in all cases: peramivir in 78.6 %, oseltamivir in 42.9 %, and zanamivir in 7.1 %; two drugs were used in each of four cases.

All patients received mechanical ventilation with endotracheal intubation. Airway pressure release ventilation (APRV) was used for mechanical ventilation in 92.9 % of cases. The causes of death were multiple organ failure (MOF) in four cases, respiratory failure in three cases, MOF and uncontrollable bleeding in one case, and concomitant respiratory and circulatory failures in one case. One of the discharged patients had respiratory sequelae.

### ECMO equipment and cannula (Tables 2, 3)

ECMO equipment manufactured by Terumo Corporation (Tokyo, Japan) was used in 11 patients (78.6 %). This equipment consists of a console, circuit, oxygenator, and centrifugal pump. The blood drainage cannula was smaller than 20 Fr. in 10 patients (71.4 %), and the maximum size was 21.5 Fr.

**Table 2:** Extracorporeal membrane oxygenation (ECMO) equipment used

Equipment	Number of cases
Console	
CAPIOX SP-101	11
Bio-Console 560	1
Stöckert SCP system	1
MERA HAP-31	1
Circuit	
CAPIOX Custom Pack	11
Unknown	3
Oxygenator	
LX or SX	9
BIOCUBE 6000	4
MERA HP Exelungprime	1
Centrifugal pump	
CX-SP45	11
COBE revolution	1
Unknown	2

CAPIOX SP-101, CAPIOX Custom Pack, LX, SX, CX-SP45 (Terumo, Tokyo, Japan), Bio-Console 560 (Medtronic, Minneapolis, MN, USA), Stöckert SCP system (SORIN Group, Germany), MERA HAP-31, MERA HP Exelungprime (Senko Medical Instrument, Tokyo, Japan), BIOCUBE 6000 (NIPRO, Osaka, Japan), COBE revolution (SORIN Group, Italy)

**Table 3:** Cannula size, approach site, and proximal position for ECMO

Drainage	Number of cases
Size (Fr.)	
18	6
19.5	4
21	3
21.5	1
Approach site	
Femoral vein	14
Proximal position	
Inferior vena cava	10
Right atrium	4

Return	Number of cases
Size (Fr.)	
12	1
15	9
16	2
16.5	1
21	1
Approach site	
Right jugular vein	12
Femoral vein	2
Proximal position	
Superior vena cava	8
Right atrium	4
Inferior vena cava	2

### ECMO therapy (Table 4)

All patients received venovenous ECMO therapy, and none required a switch to venoarterial ECMO therapy. None of the facilities had extensive experience with ECMO therapy for respiratory failure. At five facilities, ECMO was used for the first time. Six facilities had previously applied this therapy to one or two cases per year. One facility had used ECMO in at least five cases a year.

**Table 4:** ECMO therapy

	All cases (14 patients)	Survival group (5 patients)	Non-survival group (9 patients)
Ventilator days before ECMO (days)	5.0 (0.8–8.5)	3.0 (0.5–7.0)	6.0 (0.5–14.0)
Length of ECMO therapy (days)	8.5 (4.0–10.8)	9.0 (6.5–12.5)	8.0 (3.5–11.5)
Number of circuits used	2.0 (1.0–3.0)	2.0 (1.5–2.5)	2.0 (1.0–3.5)
Duration of each circuit (days)	4.0 (3.2–5.3)	5.0 (3.3–6.8)	4.0 (2.1–4.3)

Before the start of ECMO therapy, mechanical ventilation had been applied for more than 7 days in two cases from the survival group and three cases from the non-survival group (13, 15, and 20 days, respectivley). The duration of ECMO therapy was 8.5 (4.0–10.8) days, ranging from 1 day (outcome, death) to 39 days (outcome, death).

The duration of each ECMO circuit was only 4.0 (3.2–5.3) days. The ECMO circuit was renewed a total of 19 times among 10 cases. The reasons for renewal were reduced oxygenating capability owing to oxygenator failure (nine times), thrombus attachment to the oxygenator (three times), circuit obstruction with thrombus (three times), poor blood drainage flow (twice), pump head trouble (once), and hemolysis (once). The duration of ECMO therapy for the four cases that did not require circuit renewal was 6 days (outcome, survival), 4 days (death), 4 days (death), and 1 day (death).

### Adverse events associated with ECMO therapy (Table 5)

Excluding 1 patient who died on the first day of ECMO therapy, all patients developed adverse events associated with ECMO (92.9 %). Direct adverse events developed in 11 patients (78.6 %); reduced oxygenating capability owing to oxygenator failure (50 %) was the most frequent. Indirect adverse events developed in 12 patients (85.7 %). The most frequent complication was disseminated intravascular coagulation (DIC, 71.4 %). All these adverse events were associated with the coagulation and fibrinolytic system (DIC, massive bleeding, thrombus, etc.). One patient underwent a surgical procedure to achieve hemostasis.

**Table 5:** Adverse events related to ECMO therapy

Event	Number of cases (%)
Directly related to the ECMO circuit	11 (78.6)
Oxygenator failure	7 (50.0)
Blood clots	4 (28.6)
Oxygenator	3 (21.4)
Other circuit	1 (7.1)
Cannula-related problems	3 (21.4)
Pump head complications	1 (7.1)
Indirectly related to the ECMO circuit	12 (85.7)
Massive bleeding	8 (57.1)
Surgical site bleeding	4 (28.6)
Upper digestive tract hemorrhage	4 (28.6)
Cannulation site bleeding	2 (14.3)
Pulmonary hemorrhage	1 (7.1)
Hemolysis	2 (14.3)
Disseminated intravascular coagulation	10 (71.4)
Venous thrombus	2 (14.3)

## Discussion

During the 2010–2011 season in Japan, the survival rate of patients with 2009 influenza A(H1N1) severe respiratory failure following ECMO therapy was only 35.7 %. Most patients developed adverse events associated with this therapy.

Several reports have demonstrated the effectiveness of ECMO therapy for H1N1-related severe respiratory failure [2, 3, 5]. According to a report from the United Kingdom, the survival rate following ECMO therapy was 76 %, which is significantly higher than that following conventional mechanical ventilation (48 %), and thus demonstrates the effectiveness of ECMO [4]. In the present study in Japan, the survival rate was only 35.7 %, and the mortality rate was 2.5 times that predicted from the APACHE II score. The survival rate was 64 % in H1N1-related severe respiratory failure treated with mechanical ventilation without ECMO for season of 2010–2011 in Japan [8, 9].

In addition, underlying diseases were present in only two patients in this study, and none of the patients had complications involving respiratory, cardiac, or immunological disorders. Anti-influenza drugs had been used in all cases. In particular, during this season, peramivir, a drug for intravenous administration, was newly available on the market and had been used in 11 patients (78.6 %). This drug was used as a more reliable means of treatment than oseltamivir because it is less likely to cause poor absorption via the digestive tract from such problems as vomiting. This finding suggests that the lives of many of these patients could have been saved if appropriate management with ECMO had been applied.

The blood drainage cannula size is considered an important factor for maintaining appropriate flow during ECMO therapy [10]. According to a report from the ECMO Center Karolinska, blood drainage cannulas with sizes between 23 and 29 Fr. were used for patients with a median body weight of 88 kg [5]. In the present study, the drainage cannula size was <20 Fr. in 70 % of the patients, who had a median body weight of 70 kg (range, 51–90 kg) and height of 146–190 cm (estimated from body weight and BMI). According to the previous reports, the achieved ECMO blood flow rates are generally 4–5 l/min [2, 5, 6]. We did not have any ECMO blood flow data in this study, but the cannulas used for these Japanese patients appear to have been too small in diameter. The use of a blood drainage cannula with too small a diameter is more likely to cause adverse events such as inadequate flow (from poor blood drainage flow), hemolysis (due to the need for a sufficiently high pump rotation rate to achieve satisfactory flow), and a hemorrhagic tendency (caused by platelet consumption).

Recently, adverse events arising from ECMO therapy have been clearly decreasing thanks to advances in component technology and techniques [11]. However, in the present study, adverse events associated with ECMO therapy developed in all patients, except for one who died on the first day of this therapy, and the incidence of adverse events was remarkably high compared with that in previous reports [2, 4–7, 11–14]. Among other adverse events, such disorders of the coagulation and fibrinolytic system as massive bleeding, DIC, and thrombus formation, which are complications that require close attention during ECMO therapy [15], developed in most patients. Problems with the equipment and the excessively small diameter of the cannulas were probably involved in the development of many of the adverse events associated with ECMO therapy in this study. This view is supported by the observation that the duration of each circuit was only 4 days. The life of the oxygenator was extremely short, and this was a major factor in necessitating circuit renewal only 4 days after the start of use. The recommended period of use is only 6 h for the most frequently employed ECMO circuit and oxygenator in Japan, the CAPIOX Custom Pack (Terumo, Tokyo, Japan), according to its package insert (written in Japanese). The cavity of the circuit used in the present study usually had a volume of 500–600 ml. Every time the circuit was renewed, the same volume of blood was lost, and blood transfusion or intravenous fluid infusion was carried out to compensate for the discarded blood. This procedure is a major source of stress for patients. It would appear to be necessary to review the ECMO equipment used in Japan.

Factors that possibly raised the mortality rate following ECMO therapy include central nervous system injury, gastrointestinal or pulmonary hemorrhage, and renal dysfunction [16]. We found, however, no particular differences in any of these factors between the survival and non-survival groups. The maximum SOFA score during treatment was higher in the non-survival group, which reflects the tendency for a more severe disease course in the non-survival group. The only difference between the two groups is that the non-survival group included some patients who were given mechanical ventilation for a period much longer than 7 days before beginning ECMO. When started within 6 days after initiating mechanical ventilation, ECMO therapy offers a high survival rate [3, 6, 10, 11, 17–20]. It is also possible that initiation of ECMO was delayed because Japanese physicians are unfamiliar with this therapy. In addition, it seems that Japanese physicians had not understood or implemented such routine therapeutic strategies as the ELSO guidelines. Instead, a specific form of ventilation that maintained a high average airway pressure, such as APRV, was employed in many cases. Although the setting for mechanical ventilation during ECMO therapy was not sufficiently clear from the data, it appears likely that a high-pressure setting for mechanical ventilation was adopted even during ECMO therapy, and this may be one of the factors responsible for the high mortality rate. It is necessary for physicians to develop a proper understanding of the ECMO treatment strategy.

The survival rate of adults with severe respiratory failure following ECMO therapy is reported to be usually 61 % [15]. It has also been reported that when ECMO therapy is applied to patients with severe respiratory failure, transfer to a central facility, such as an ECMO center, is likely to yield better outcomes [4–6, 11, 12, 14]. During the 2009–2010 season, ECMO therapy was applied to 16 patients with H1N1-related severe respiratory failure in Sweden; 13 (81 %) of these patients were transferred to the ECMO Center Karolinska, and the result was successful weaning from ECMO in all cases [5]. In Italy, establishment of the ECMO network resulted in a high survival rate [6]. Both the effectiveness of ECMO therapy for H1N1-related severe respiratory failure and treating many cases at the central facility were reportedly major factors contributing to the high survival rate [4]. The facilities in Japan have very little experience with ECMO therapy for patients with severe respiratory failure. At most Japanese facilities in the present study, ECMO for severe respiratory failure had been applied to only one or two respiratory failure cases a year or even less frequently; about half of the facilities had no previous experience with this therapy. H1N1-related severe respiratory failure has a high probability of recovery in response to ECMO. Thus, adopting ECMO therapy should be given due consideration. However, because the number of patients with this condition is not particularly large, transferring patients to central facilities for this therapy is anticipated to improve treatment outcomes because the physicians at such centers can gain experience through dealing with a larger number of cases. ECMO may also be indicated for H5N1 (avian influenza), an outbreak of which is now a great concern. A recent report has shown that ECMO should be performed at centers with high case volumes, established protocols, and clinicians who are experienced in its use [11]. Facilities serving as centers for this therapy should be established in Japan as soon as possible.

The present study has a limitation in that the survey did not cover all patients who received ECMO therapy. According to a report by the Ministry of Health, Labour and Welfare of Japan, there were 15 deaths among the adults who were given ECMO therapy (the number of survivors has not been made public) [21]. In the present study, 9 of the patients died, which would suggest that more than half of all Japanese patients who received ECMO therapy were covered by this survey.

The survival rate for patients with H1N1-related severe respiratory failure following ECMO therapy in the present study was very low. However, this result does not refute the effectiveness of ECMO therapy for H1N1-related severe respiratory failure; the result is instead attributable to the lack of experience and lack of preparedness of Japanese facilities to provide ECMO therapy. To improve the outcomes of ECMO therapy not only in Japan but also in other countries inexperienced with ECMO therapy, efforts should be made along the following lines: (1) supply ECMO equipment suitable for treatment of severe respiratory failure; (2) promote a full understanding of the ECMO treatment strategy by physicians and other medical staff; and (3) transfer patients to central facilities established for this therapy.
